# BioAFMviewer: An interactive interface for simulated AFM scanning of biomolecular structures and dynamics

**DOI:** 10.1371/journal.pcbi.1008444

**Published:** 2020-11-18

**Authors:** Romain Amyot, Holger Flechsig

**Affiliations:** 1 Department of Mathematical and Life Sciences, Graduate School of Science, Hiroshima University, Higashi-Hiroshima, Hiroshima, Japan; 2 Nano Life Science Institute (WPI-NanoLSI), Kanazawa University, Kakuma-machi, Kanazawa, Ishikawa, Japan; Hebrew University of Jerusalem, ISRAEL

## Abstract

We provide a stand-alone software, the BioAFMviewer, which transforms biomolecular structures into the graphical representation corresponding to the outcome of atomic force microscopy (AFM) experiments. The AFM graphics is obtained by performing simulated scanning over the molecular structure encoded in the corresponding PDB file. A versatile molecular viewer integrates the visualization of PDB structures and control over their orientation, while synchronized simulated scanning with variable spatial resolution and tip-shape geometry produces the corresponding AFM graphics. We demonstrate the applicability of the BioAFMviewer by comparing simulated AFM graphics to high-speed AFM observations of proteins. The software can furthermore process molecular movies of conformational motions, e.g. those obtained from servers which model functional transitions within a protein, and produce the corresponding simulated AFM movie. The BioAFMviewer software provides the platform to employ the plethora of structural and dynamical data of proteins in order to help in the interpretation of biomolecular AFM experiments.

## Author summary

Nowadays nanotechnology allows to observe single proteins at work. Under atomic force microscopy (AFM), e.g., their surface can be rapidly scanned and functional motions monitored, which is of great importance for applications in all fields of Life science. The analysis and interpretation of experimental results remains however challenging, because the resolution of obtained images or *molecular movies* is far from perfect. On the other side, high-resolution static structures of most proteins are known and their conformational dynamics can be computed in molecular simulations. This enormous amount of available data offers a great opportunity to better understand the outcome of resolution-limited scanning experiments. Our software provides the computational package towards this goal. The BioAFMviewer computationally emulates the scanning of any biomolecular structure to produce graphical images that mimic the outcome of AFM experiments. This makes the comparison of all available structural data and computational *molecular movies* to AFM results possible. We demonstrate that simulated AFM images are of great value to facilitate the interpretation of high-speed AFM observations. With its versatile interactive interface and rich functionality, the BioAFMviewer provides a convenient platform for the broad Bio-AFM community to better understand experiments.

This is a *PLOS Computational Biology* Software paper.

## Introduction

*Seeing is believing* paraphrases the holy grail of molecular biophysics—the imaging of molecular processes which establish Life at the nanoscale. At the single protein level, this task is particularly challenging, due to the tininess of structures and the rapid timescales of functional conformational motions.

Atomic force microscopy (AFM) experiments can provide high-resolution images of biological structures and probe functional properties at the single-molecule level, e.g. [1–5]. With the development of the high-speed atomic force microscopy (hs-AFM) technique, the breakthrough of visualizing functionally relevant motions became possible. Hs-AFM allows to observe conformational dynamics in proteins under physiological conditions by rapidly scanning over the protein surface and imaging its shape [6–10]. To date, hs-AFM is a leading method to observe dynamical processes in proteins and its success has been evidenced in a plethora of studies [11–14].

Like any other experimental approach, hs-AFM has also limitations. The temporal resolution is limited by the rate with which *protein movies* can be recorded (typically less than 10 frames per second). The spatial resolution allows to resolve well the motions of domains but is typically not high enough to visualize changes of individual structural elements which may be of functional importance. Another drawback is the control of the molecular orientation of proteins deposited on the surface. Furthermore, in a single experiment observation proceeds from a fixed viewpoint, generally limiting the information on the coupling of motions between opposite regions within a protein.

By improving technological aspects of hs-AFM, those drawbacks can be overcome only up to a certain extent. A promising strategy to help in the interpretation and understanding of measurement results may consist in a complementary approach, taking into account the plethora of available structural data and computational modelling of biomolecular systems.

The static structures of many biological macromolecules are known. This data is deposited in the Protein Data Bank (PDB) [15, 16] in a well-defined format, providing the spatial coordinates of atomic positions in a PDB file. On the other side, functional conformational dynamics in proteins, such as the motions underlying e.g. ligand-induced transitions, can often be obtained from modelling servers, e.g. [17–19].

A comparison of the wealth of both static and dynamical protein data to AFM experiments is therefore possible. This, however, requires the development of suitable computational tools. We provide a stand-alone software which makes an important step in that direction. The BioAFMviewer transforms the molecular representation of any structure given in the PDB file format into the corresponding AFM graphics by performing simulated scanning. The software implements a high degree of usability. A versatile molecular viewer allows the visualization of molecular structures and control over their orientation, while synchronized scanning with variable spatial resolution and tip-shape geometry generates the corresponding AFM graphics. Obtained results can be conveniently exported as images and movies. We demonstrate the applicability of the BioAFMviewer by comparing simulated AFM images with snapshots of selected hs-AFM experiments of proteins.

## Design and implementation

We focus here on a description of the general workflow of the BioAFMviewer, from the protein structure displayed in the integrated molecular viewer to the corresponding simulated AFM graphics.

### Molecular viewer of protein structures and movies

The software can process static structures and molecular movies as long as they are provided in the standard PDB file format (see www.wwpdb.org/documentation/file-format). An integrated molecular viewer visualizes the three-dimensional molecular structure as encoded in the loaded PDB file. All atoms are displayed in the Van der Waals (VdW) representation, as spheres with the radii each given by the specific VdW radius of the atom type. The orientation of the loaded protein structure can be arbitrarily changed through rotations and zooming within the viewer window. The instantaneous molecular structure is translated into the corresponding AFM graphics by performing synchronized simulated scanning, and the results are displayed in a separate window. We next describe how simulated scanning is implemented.

### Simulated AFM images

Generally in an AFM experiment, the biomolecule (sample) deposited on a solid surface (stage) is scanned by the tip along a defined two-dimensional grid and tip-sample interactions provide information on the height of the sample relative to the stage. To visualize the output, this information is then processed to produce graphical images of the sample shape with color-coded heights along the scanning grid.

**Fig 1:**
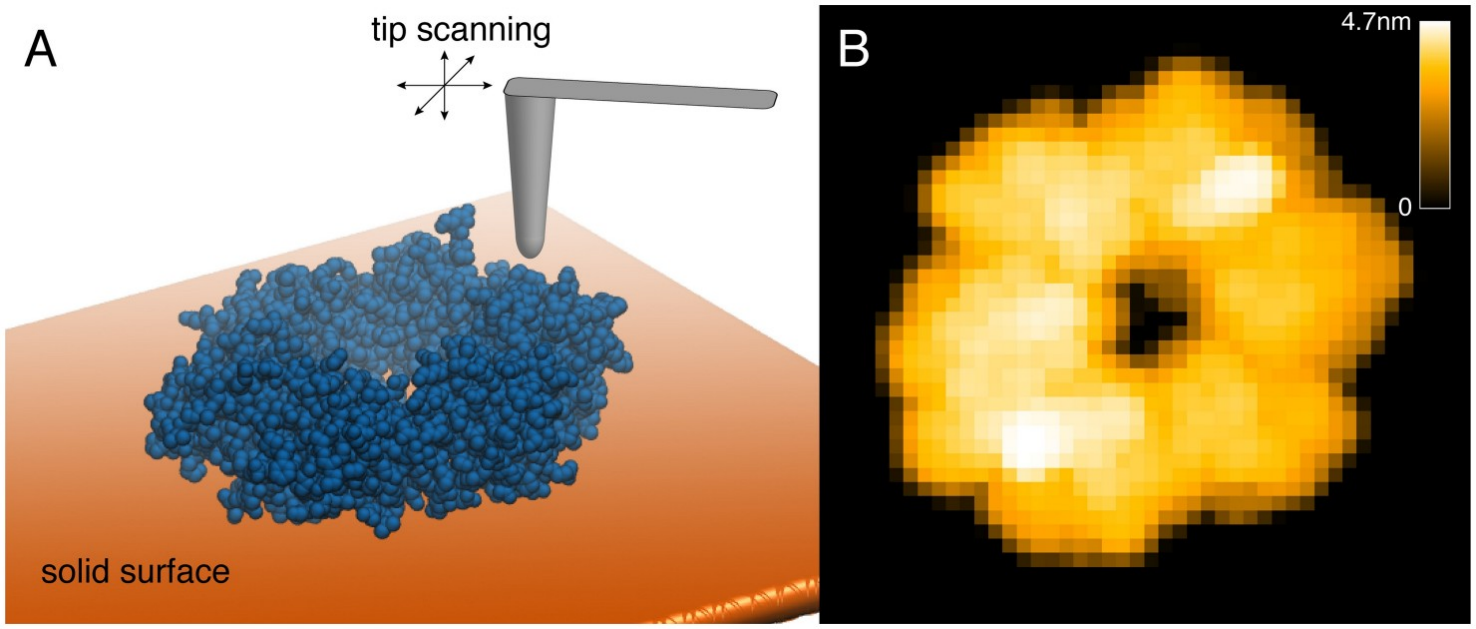
Simulated AFM scanning and graphics. A) Schematic illustration of simulated scanning. A solid cone-shaped tip is scanning over the protein structure which is deposited on a virtual solid surface. B) The corresponding generated two-dimensional AFM graphics with the colors encoding the height of the protein shape (see colorbar) with respect to the surface for all cells of the scanning grid.

While in experiments tip-sample interactions during scanning are very complex, simulated scanning shall provide an approximate method to translate the set of atom coordinates in a biomolecule into the height map that would roughly correspond to the outcome of an AFM experiment. The method we apply for simulated scanning relies on non-elastic hard collisions of the tip with the atomic VdW spheres of the biomolecule, and has been previously employed [8, 20, 21]. The tip is assumed to have a cone-like shape with a probe sphere at its narrow end. Its geometry is characterized by the cone half-angle *α* and the probe sphere radius *R*. To determine the height map of the biomolecule in a given orientation, the corresponding molecular structure is scanned by the tip over a two-dimensional grid with the step size *a*. A virtual surface determined for the actual orientation provides the corresponding reference frame. For each cell within the scanning grid, the height relative to that surface is determined from the collision condition of the tip shape with the VdW spheres of all atoms. In S1 Fig we provide a schematic representation. For a detailed mathematical formulation of this scanning procedure, we refer to the Supplementary Information. In Fig 1 we provide an illustration of the simulated scanning process together with the produced AFM graphics of an example protein (see S1 Text).

Simulated scanning in the BioAFMviewer operates synchronous with the instantaneous orientation of the biomolecule displayed in the viewer window. To demonstrate this functionality we provide video recordings of a medium size protein and of a very large protein complex. S1 Video shows simulated scanning of the SARS-CoV-2 RNA polymerase protein (∼8500 atoms). The simulated AFM graphics of the protein in its actual orientation appears in perfect synchronization using a standard laptop computer (see S1 Text). In S2 Video we considered the large GroEL-GroES chaperone complex (∼60.000 atoms), which has been previously investigated using hs-AFM microscopy [22]. Though this case is computationally much heavier, a smooth simultaneous observation of both molecular and simulated AFM graphics is available. In the orientation in which the GroEL-GroES protein complex is scanned in the side perspective, its bullet shape formed by the GroEL 14-mer and the GroES cap is seen, resembling previous hs-AFM images [22].

**Fig 2:**
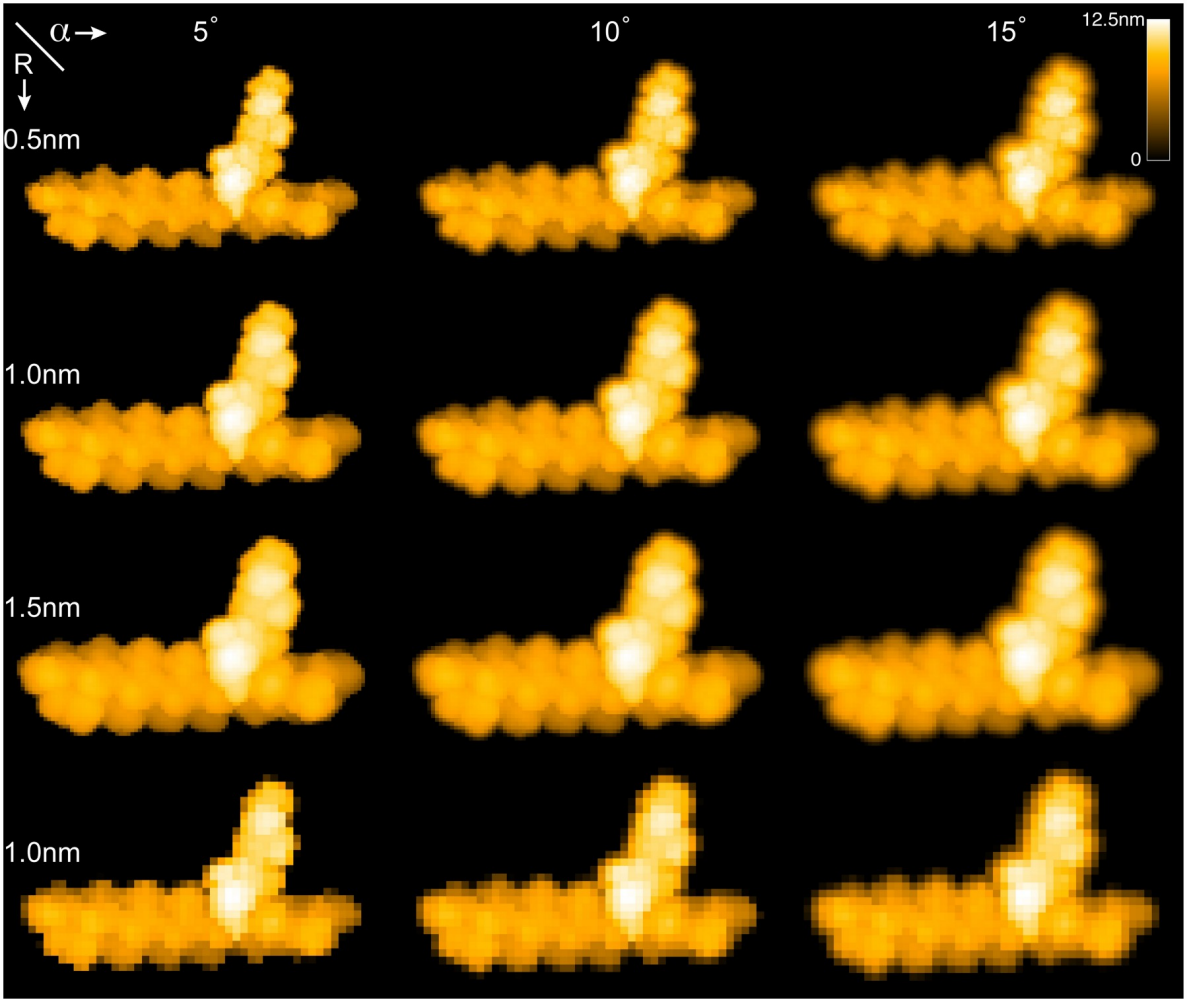
Simulated AFM scanning with various parameters. Simulated AFM images for the acto-myosin complex are shown for different scanning parameters. Rows display images generated with different tip-shape radii, while columns show them obtained with changing tip-shape angle. The scanning resolution of images in the first three rows was 0.5nm and that of the fourth row was 1.0nm.

The BioAFMviewer enables simulated scanning with variable parameters of the tip shape geometry and of the spatial resolution of the scanning grid. All parameters can be conveniently changed in switching panels of the *Assistant window*, while the updated AFM graphics is synchronously displayed. In Fig 2 we provide a demonstration of how different scanning parameters affect the generated AFM graphics. We have considered the structure of the acto-myosin complex in a fixed orientation and simulated scanning with different values of the cone-half angle *α* = 5°, 10°, 15° and of the probe sphere radius *R* = 0.5nm, 1.0nm, 1.5nm.

As expected, changes in the tip-shape geometry significantly affect the produced simulated AFM image. Increasing e.g. the probe radius *R* results in coarser scanning of the molecular surface, effectively producing smoother AFM images which however increasingly suffer from resolving structural details (see rows in Fig 2). Changes in the tip cone-half angle *α* have a pronounced effect mainly visible at the protein boundaries or intramolecular cavities. Increasing it results in a larger blurring of the corresponding areas in the simulated images (see columns in Fig 2). The spatial resolution of scanning is controlled by the step size of the tip along the scanning grid; increasing it will obviously produce more pixelated simulated AFM images as we demonstrate in Fig 2 (bottom row).

### Simulated AFM molecular movies

Conformational dynamics in biomolecules can be readily obtained from computational simulations. In particular coarse-grained protein models are able to capture slow domain motions and allow to monitor functional cycles of proteins in a *molecular movie* [23–25]. Such movies may for example visualize conformational changes along functional transitions within a protein, using the vast amount of structural data. They may also represent large-amplitude collective domain motions in a protein computed from normal modes of its structure, as they typically well approximate functional transitions. Often, the underlying computation is automatized and performed by modelling servers which provide user-friendly interfaces, allowing even non-experts to obtain protein *molecular movies* without high-end resources.

A comparison between *computational molecular movies* obtained from simulations with *molecular movies* recorded in hs-AFM experiments is therefore available. The BioAFMviewer software takes that into account by providing simulated AFM graphics of molecular movies provided in the PDB format. In this case simulated AFM images are generated frame by frame from the molecular representation of the loaded movie with the same previous described versatile functionality. For the user-selected parameter set, both the movie in the molecular representation and the corresponding simulated AFM movie are synchronously displayed. In the Results section we provide an example.

## Results

In the BioAFMviewer, simulated scanning with variable tip shapes and spatial resolution, together with the convenient control over the corresponding parameters allows to compare available data of biomolecules to experimental observations. As a demonstration, we show how simulated AFM graphics produced from static PDB structures of proteins can help in the interpretation of high-speed AFM experimental data.

### Comparison of simulated AFM graphics to high-speed AFM experiments

We demonstrate how the BioAFMviewer software can be employed in the interpretation of experimental hs-AFM images. Examples for three different protein machines from previously published high-impact publications [8, 20, 26] are provided. Our workflow is as follows. For the investigated proteins, we select PDB structures which are related to the experimental situation, e.g., possibly taking into account structures complexed with other molecules (such as ligands). When the selected PDB structure is loaded into the BioAFMviewer, the VdW representation and the corresponding simulated AFM image is shown. Then, the scanning parameters are adjusted and the orientation of the protein is changed to obtain a simulated AFM image which resembles the experimental target AFM snapshot. The obtained images can be saved on-click in the *Assistant Window*.

**Fig 3:**
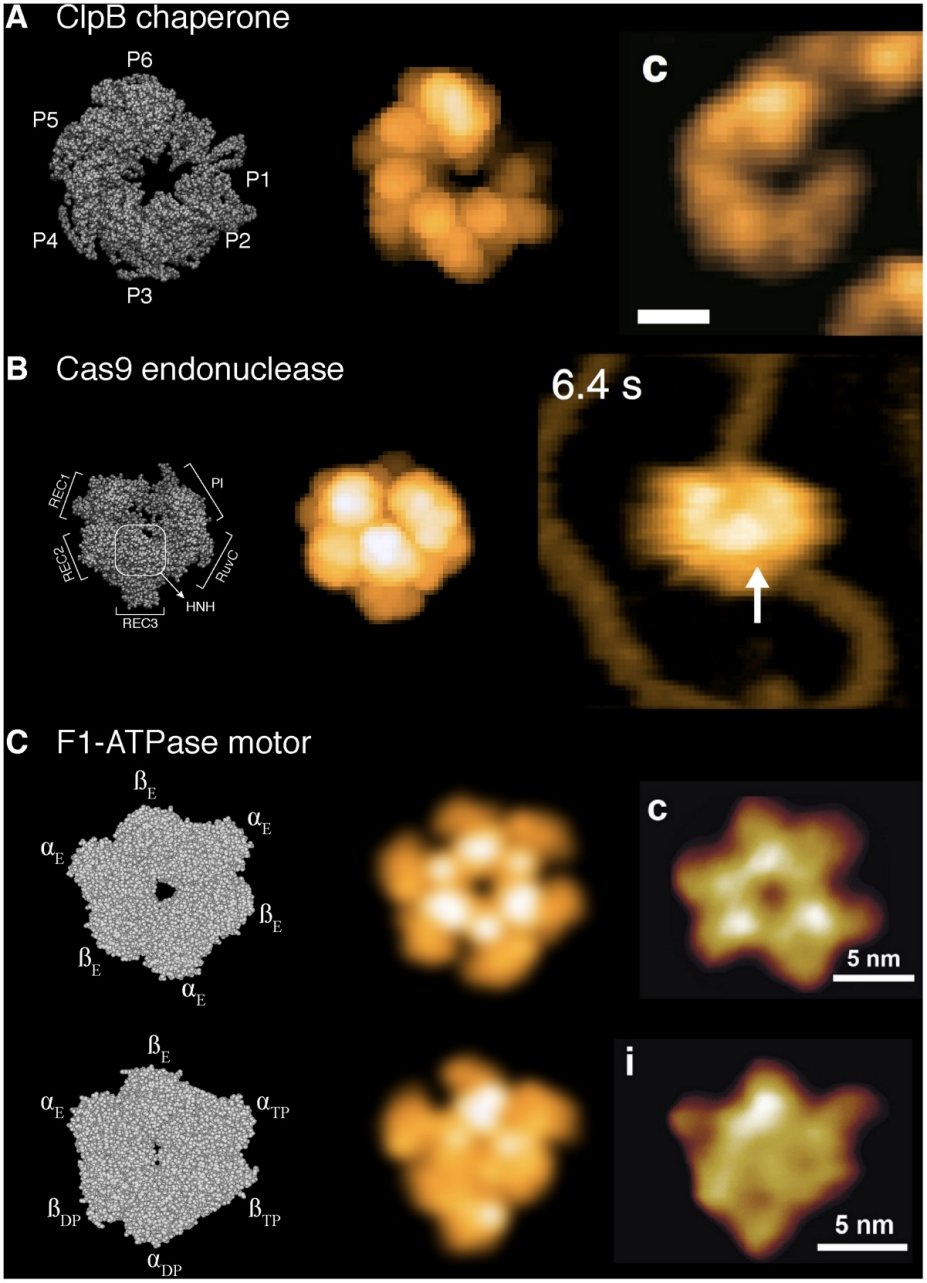
Comparison of simulated AFM graphics to high-speed AFM experiments. Static protein structures and their corresponding simulated AFM graphics are compared to snapshots from hs-AFM experiments. The selected examples are the ClpB chaperone (A), the Cas9 endonuclease (B), and the F1-ATPase molecular motor (C). In all panels the left image shows the molecular protein structure with the individual domains indicated, the middle image shows the corresponding simulated AFM graphics, and the representative hs-AFM snapshots are shown at the right. The hs-AFM figures are adopted from references [20] (ClpB), [26] (Cas9), and [10] (F1-ATPase). See main text for further explanations, and S1 Text for details on the used PDB structures.

The selected examples are shown in Fig 3. The first is the ATP-dependent protein disaggregation machine ClpB, whose functional activity has been recently investigated in hs-AFM experiments [20]. In Fig 3 (panel A) we show the ClpB molecular structure, the corresponding simulated AFM image, and the experimental image. The similarity of the simulated and real AFM image is remarkable. The molecular structure in that exact orientation together with the simulated AFM image can therefore validate the hs-AFM observation. Furthermore, it allows to disambiguate the arrangement of protomers P1 to P6 in the hs-AFM image of the hexameric ClpB machine, and can provide important information to deduce its mechanism from hs-AFM data.

The second example is the gene scissor related CRISPR-associated protein 9 endonuclease (Cas9). Hs-AFM experiments provided great new insights into the dynamic operation of the CRISPR-Cas9 system [26]. In Fig 3 (panel B) we demonstrate how simulated imaging can help to identify the structure of domains in resolution-limited snapshots of hs-AFM movies of this protein. From the detailed molecular presentation of this protein (Fig 3B, left) and the corresponding obtained simulated AFM image (Fig 3B, middle), we can clearly label functional domains roughly seen in the hs-AFM image and clarify their orientation with respect to the bound DNA strand (Fig 3B, right).

The third example is the ring-shaped F1-ATPase molecular machine of the ATP synthase motor. In a seminal work Uchihashi et al. have used hs-AFM to demonstrate that this machine can perform ATP-dependent rotary conformational motions even in the absence of the central rotor shaft [8]. We have used the BioAFMviewer to produce simulated AFM images of the F1 ring structure in absence of nucleotides, and of one with ATP-analogs and ADP bound to different domains in the ring. Our aim was to compare them to the experimentally obtained averaged AFM images of the F1 ring without nucleotides, and under saturating conditions of an ATP-analog, provided from the original publication. The results are shown in Fig 3, panel C. The molecular representation of the chosen PDB files in their target orientation is shown in Fig 3C (left). The corresponding produced simulated AFM images were filtered by a Gaussian blur to take into account experimental image averaging in this case. The final simulated images (Fig 3C, middle) are in great agreement with experimental observations. They confirm the symmetric shape of the F1 ring under experimental conditions without any nucleotides, as well as the asymmetric shape in the nucleotide-complexed case (Fig 3C, right). The latter case demonstrates how simulated imaging in principle allows to identify the nucleotide state of individual domains in experimental images of large protein complexes, by comparing domain shapes between simulated and experimental AFM images. We note that in the original publication simulated image construction was already employed, using however a non-publicly available software. Our goal was to demonstrate that the BioAFMviewer software can readily produce such simulated images with ease, not employing additional dependencies on other commercial software, and therefore providing a public platform available to the broad community of AFM experimentalists.

Within the current workflow, the comparison of simulated AFM graphics to experimental AFM images is rather qualitative. While for the given examples convincing visual agreement is demonstrated, we also provide a quantitative score to evaluate the similarity between simulated and experimental AFM results in terms of their image correlations (see S1 Text). A similar method has been employed in a recent application of flexible fitting protein structures to AFM templates [21]. The results of our analysis are shown in S2 Fig. For all examined protein examples from Fig 3 correlations are larger than 0.9, which substantiates the obtained visual agreement. We discuss future challenges and prospects of integrating quantitative comparison between simulated and experimental AFM data in the last section.

In the Discussion section we also explain the drawbacks of simulated scanning and mention limitations in the comparison of computational AFM graphics to experimental data.

### Simulated AFM molecular movies

For the purpose of demonstration, we have considered a molecular movie of the GroEL chaperone complex. It monitors conformational motions which approximately describe the close-to-open transition of the 7-mer ring structure which is related to interactions with ATP molecules in the individual monomers of this protein machine. The feasible pathway of conformational transition was computed by the iMODS server based on the iterative analysis of normal modes [27]. S3 Video shows several cycles of the movie in the molecular VdW representation and the synchronous AFM graphics side by side. Two perspectives are provided, corresponding to scanning the protein in the side view orientation and the top view orientation, respectively. In the side perspective, the height protrusions in the simulated AFM movie can be nicely assigned to the individual functional transition within two neighboring subunits of the ring (see S3 Fig). In the simulated AFM movie displayed in top view perspective, iris-like shape changes are reproduced and opening motions of domains in the ring can be clearly seen from changes in their protrusion heights.

The functionality of transforming molecular movies into their corresponding AFM graphics by the BioAFMviewer software provides the platform to generate *simulated AFM experiments* which can be compared to real AFM experiments in future applications.

## Discussion

The BioAFMviewer is the first publicly available software which provides simulated AFM graphics of biomolecular structures and movies of their conformational motions. Our emphasis is to provide a platform which combines an user-friendly interface with the functionality required to make comparison to experiments available. Applications to interpret images of high-speed AFM experiments of proteins demonstrate the importance of the BioAFMviewer to complement experimental observations. Moreover, *simulated AFM experiments* become in principle possible, which will facilitate the interpretation of resolution-limited experimental observations.

### Limitations of simulated scanning

Simulated scanning is an approximation which in many aspects represents an idealization of the complex AFM scanning procedure. First of all, it should be noted that PDB protein structures are often obtained under artificial conditions (i.e. using crystallization), whereas AFM experiments are conducted with proteins in solution. The main facet, however, is that proteins and other biomolecules are soft material which undergo conformational motions to execute their function. When observed with AFM, additional deformations of their structures arise due to interactions with the surface and the perturbations which are constantly applied with the AFM tip. To resolve those interactions is beyond the intention of the employed mechanism of simulated scanning, which is to provide a computationally efficient method of producing AFM graphics in synchronous operation with the molecular viewer. Simulated scanning is also free of any experimental noise which inherently leaves a background in AFM images.

The described drawbacks present limitations when comparing simulated AFM graphics to experimental observations. It should not be expected, for example, that simulated scanning would reproduce the magnitude of shape heights seen in an AFM experiment. For the studied examples we indeed found overall larger height scales of protein shapes from simulated scanning when comparing with those of hs-AFM images, which is due to the applied non-elastic collision method of the tip with the sample. One can obviously also not expect to obtain precise agreement between computationally scanned contours of protein domains and those scanned e.g. by hs-AFM.

Despite all simplifications, simulated scanning can very well reproduce the shape of individual protein domains and resolve their relative protrusions seen in hs-AFM experiments. For the studied examples the similarity of simulated and hs-AFM graphics is remarkable. Furthermore excellent quantitative agreement based on image correlation analysis is obtained.

### Availability and future directions

We report here the BioAFMviewer in its present version. The software is already used by many AFM groups worldwide, and we are getting valuable response which helps to further advance the platform. Based on the feedback received so far, we are expecting the BioAFMviewer software to become a standard platform used by the broad community of Bio-AFM experimentalists. Beyond that, it also provides the interface for researchers from the fields of computational biology and bioinformatics to foster their interdisciplinary collaborations. The BioAFMviewer software is available for download on the www.bioafmviewer.com website, where we also provide a discussion channel. All software updates will be made available there.

The upcoming improvements of the software will focus on implementing tools which allow to extend comparison of simulated and experimental AFM images to a quantitative level. As we have already demonstrated, the image correlation can provide a suitable scoring function to quantify the overall agreement between simulated and experimental AFM data. A challenge is to incorporate an automatized procedure to detect optimal fitting of simulated AFM graphics to the experimental template.

## Supporting information

S1 Text In the Supporting Text we provide all information required to reproduce the results shown in this article. Supporting Figures (S1, S2 and S3 Figs) and Videos (S1, S2 and S3 Videos) are available.
(PDF)

S1 Fig Simulated AFM scanning. A) The cone-shaped tip with its geometric parameters and the tip-structure hard collision method to generate simulated AFM images are illustrated. The molecular structure is shown in the VdW representation and the virtual sample surface determined for the given scanning orientation is indicated by the dark gray bar. B) The size of the scanning area for a given scanning orientation is shown. The step size along the scanning grid is denoted by *a*.
(TIF)

S2 Fig Image comparison of simulated and experimental hs-AFM graphics. For the examples shown in main text Fig 3, the similarity between simulated and hs-AFM images is quantified by means of their pixel intensities. Only defined regions of interest are displayed in grayscale. Left images show the simulated AFM graphics, middle images the hs-AFM graphics, and right images provide corresponding cleaned versions for more realistic comparison. In all cases, the correlation value *C* of simulated and experimental images is given (see S1 Text).
(TIF)

S3 Fig Simulated AFM molecular movies. Snapshots from the molecular movie (S3 Video) of the GroEL complex in the closed and open state (A,B). Simulated AFM images and the corresponding molecular representation are shown side by side. In the VdW representation two adjacent domains in the 7-mer ring are highlighted by red color. The functional change in their shape is nicely resolved in the height protrusions of the simulated images.
(TIF)

S1 Video Simulated scanning of the SARS-CoV-2 RNA polymerase protein. For arbitrarily changed protein orientations the molecular representation and the corresponding simulated AFM images are shown side by side.
(MP4)

S2 Video Simulated scanning of the GroEL-GroES chaperone complex. For arbitrarily changed protein orientations the molecular representation and the corresponding simulated AFM images are shown side by side.
(MP4)

S3 Video Simulated AFM molecular movie. Functional ATP-related conformational motions in the GroEL chaperone ring from the closed to the open state. Several cycles are displayed in the molecular VdW representation and the synchronous AFM graphics side by side. Two perspectives are provided, corresponding to scanning the protein complex in the side view (top row) and top view orientation (bottom row), respectively.
(MP4)
