# Supplementary material for: BioAFMviewer: An interactive interface for simulated AFM scanning of biomolecular structures and dynamics
Source: PLoS Comput Biol. 2020 Nov 18;16(11):e1008444. doi: 10.1371/journal.pcbi.1008444 (PMC7710046; doi:10.1371/journal.pcbi.1008444)
Supplement: S1 Text — Supporting Figures (S1, S2 and S3 Figs) and Videos (S1, S2 and S3 Videos) are available. (PDF) [file pcbi.1008444.s001.pdf]

BioAFMviewer: an interactive interface for simulated AFM scanning of biomolecular structures and dynamics – Supporting Information

Romain Amyot[1,2] **and Holger Flechsig**[3,*]

[1]Department of Mathematical and Life Sciences, Graduate School of Science, Hiroshima university, 1-3-1 Kagamiyama, Higashi-Hiroshima, Hiroshima 739-8526, Japan
[2]current affiliation: Adhesion and Inflammation Lab (LAI), Aix-Marseille University, 163 Av. de Luminy, 13009 Marseille, France
[3]Nano Life Science Institute (WPI-NanoLSI), Kanazawa University, Kakuma-machi, Kanazawa, Ishikawa 920-1192, Japan
[*]flechsig@staff.kanazawa-u.ac.jp

Supporting Text

Here we provide all information required to reproduce the results shown in the article. Supporting Figures (Figs S1, S2, and S3) and Videos (Videos S1, S2, and S3) are available.

PDB structures and scanning parameters

For the illustration of simulated scanning in Fig 1 we have used the crystal structure of the DNA polymerase III sliding clamp (PDB 3P16, chains A and B). Scanning parameters were $a = 0.3$nm, $\alpha = 10^\circ$ and $R = 0.5$nm.

For the video demonstration of the BioAFMviewer we have used the cryo-EM structure of the SARS-CoV-2 RNA polymerase (PDB 6M71) and the crystal structure of the GroEL-GroES chaperone complex (PDB 1SX4). The scanning parameters were $a = 0.5$nm, $\alpha = 10^\circ$ and $R = 0.8$nm, and $a = 1$nm, $\alpha = 10^\circ$ and $R = 1$nm, respectively. In both video examples the *variable scale mode* of color encoding was employed (see Manual for further explanations).

To demonstrate the effect of tip-shapes on the simulated AFM images we have used the electron microscopy structure of the muscle acto-myosin complex (PDB 1M8Q). Only a single myosin protein was considered (chains P,Q,R). The scanning parameters are given in the main text figure caption.

For comparison to the ΔN-TClpB hs-AFM image, we have simulated an image of the cryo-EM structure of the homologue Hsp104 in the ATP state (PDB 5KNE). From the initial conformation, the N-terminal domains have been truncated. Scanning parameters were $a = 0.4$nm, $\alpha = 10^\circ$ and $R = 1$nm. The color window corresponded to $6-11$nm. In the case of the Cas9-RNA-DNA experimental hs-AFM image, we have used the crystal structure of the Cas9-RNA-DNA complex (PDB 4OO8, chain A; guide RNA and target DNA has been omitted) to produce the simulated AFM image. Scanning parameters were $a = 0.35$nm, $\alpha = 10^\circ$ and $R = 1$nm. The selected color window corresponded to $0-7.8$nm. For the rotorless F1-ATPase hs-AFM case, we have used the crystal structure of the nucleotide-free ring (PDB 1SKY, ring assembly) and the crystal structure of the F1 ring with bound nucleotides (PDB 1BMF, without the rotor γ-subunit) to generate simulated images. Scanning parameters were $a = 0.3$nm, $\alpha = 10^\circ$ and $R = 1$nm. The selected color window corresponded to $7.5-10$nm, and $7.5-9.7$nm, respectively.

For the simulated AFM movie of the GroEL functional transition (Video 3) the scanning parameters were $a = 0.5$nm, $\alpha = 10^\circ$ and $R = 1$nm. For the side-view perspective the colorbar was customized to the range between $0-14$nm; that for the top-view perspective was between $0-9$nm. It should be noted that for the GroEL case the molecular movie contained only the alpha-carbon atoms of amino acid residues. Therefore, only a coarse-grained molecular structure could be considered in simulated scanning.

Comparison of simulated and experimental AFM graphics

The similarity between a simulated and an experimental AFM image can be quantified by analyzing pixel intensities of both images and computing the correlation

$$C = \frac{\sum_{i,j}\left(I_{ij}^{\text{sim}} - \bar{I}^{\text{sim}}\right)\left(I_{ij}^{\text{exp}} - \bar{I}^{\text{exp}}\right)}{\sqrt{\left(\sum_{i,j}\left(I_{ij}^{\text{sim}} - \bar{I}^{\text{sim}}\right)^2\right)\left(\sum_{i,j}\left(I_{ij}^{\text{exp}} - \bar{I}^{\text{exp}}\right)^2\right)}}. \quad (1)$$

Here, the summation is performed over all image pixels. I_{ij}^{sim} (I_{ij}^{exp}) is the intensity value of the pixel at position (i, j) in the simulated (experimental) image, and $\bar{I}^{\text{sim}}$ ($\bar{I}^{\text{exp}}$) denote average pixel intensities.

Software availability

The BioAFMviewer software is currently available for the Windows operation system. A compilation for Linux and the Mac OS system is under preparation. The package is available for download at the www.bioafmviewer.com website. A manual with detailed step-by-step instructions on how to efficiently use the BioAFMviewer is available. It also explains how the obtained results can be conveniently exported as image files and movies.

Benchmark details

The BioAFMviewer performance was tested on standard laptop machines. 1) a Lenovo-ThinkPad with Intel(R) Core(TM) i5-7200U CPU @ 2.50GHz 2.71GHZ (2 cores and 8 GB RAM) and 2) a Lenovo-ThinkPad with Intel(R) Core(TM) i7-8850H CPU @ 2.60GHz 2.59 GHz (6 cores and 64 GB RAM).

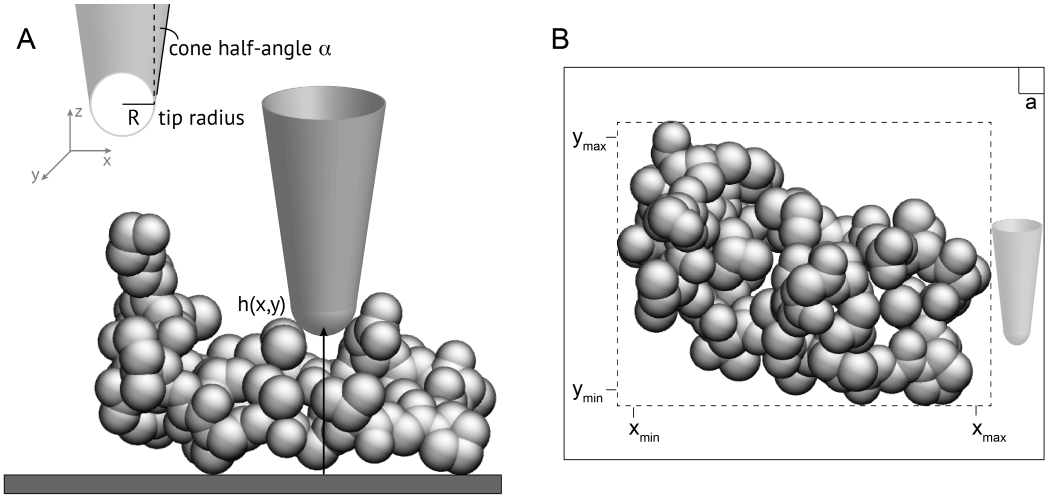

S1 Fig. Simulated AFM scanning. A) The cone-shaped tip with its geometric parameters and the tip-structure hard collision method to generate simulated AFM images are illustrated. The molecular structure is shown in the VdW representation and the virtual sample surface determined for the given scanning orientation is indicated by the dark gray bar. B) The size of the scanning area for a given scanning orientation is shown. The step size along the scanning grid is denoted by *a*.

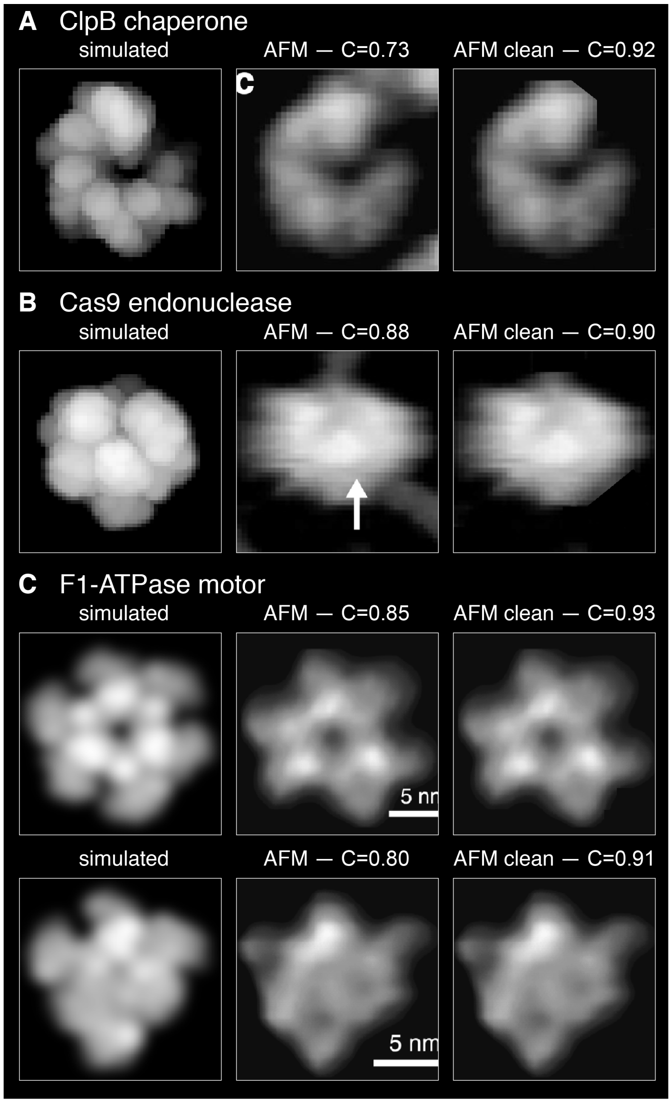

S2 Fig. Image comparison of simulated and experimental hs-AFM graphics. For the examples shown in main text Fig 3, the similarity between simulated and hs-AFM images is quantified by means of their pixel intensities. Only defined regions of interest are displayed in grayscale. Left images show the simulated AFM graphics, middle images the hs-AFM graphics, and right images provide corresponding cleaned versions for more realistic comparison. In all cases, the correlation value *C* of simulated and experimental images is given (see S1 Text).

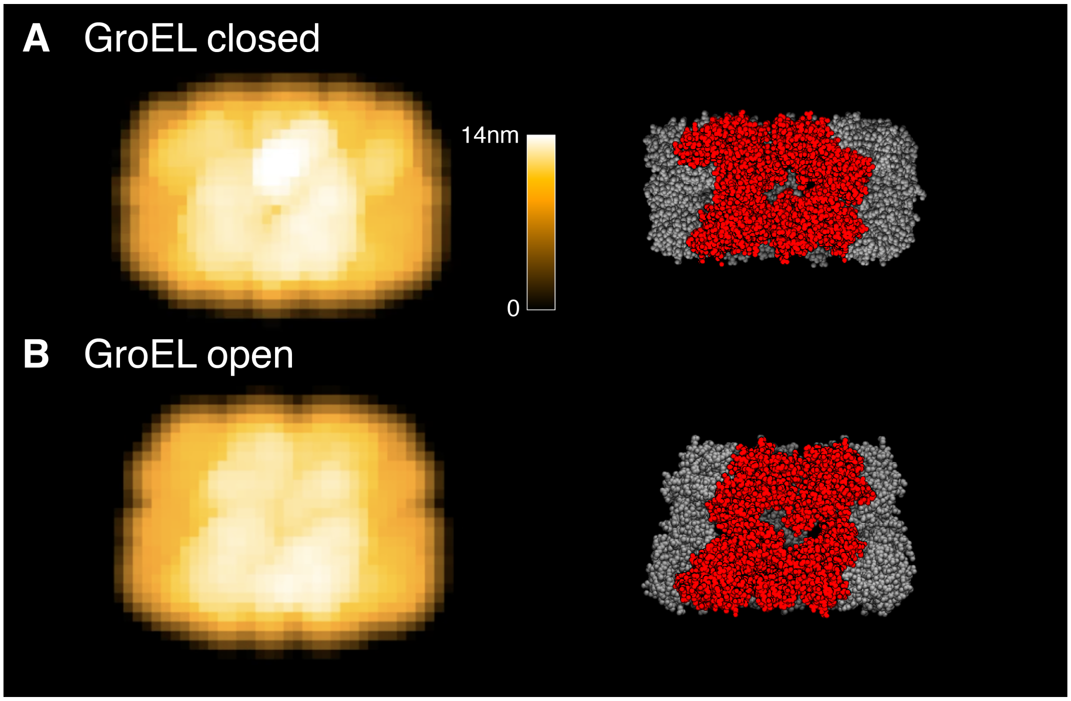

S3 Fig. Simulated AFM molecular movies. Snapshots from the molecular movie (S3 Video) of the GroEL complex in the closed and open state (A,B). Simulated AFM images and the corresponding molecular representation are shown side by side. In the VdW representation two adjacent domains in the 7-mer ring are highlighted by red color. The functional change in their shape is nicely resolved in the height protrusions of the simulated images.

Mathematical aspects of tip-sample collision

1 Shape of the tip

The tip is composed of a cone characterized by a cone half-angle α and a sphere of radius r. The sphere is placed into the cone in such a way that the centre S of the sphere is aligned to the vertex V of the cone and that the sphere intersects with the cone along a circle (see Figure 1). The cone region below the sphere (see shaded area in Figure 1) does not contribute to the tip shape. To calculate the collision between the tip and a spherical atom, we calculate both the collision between the spherical part of the tip and the sphere of the atom, and the collision between the conic part of the tip and the spherical atom. In the latter case, however, we discard collisions with the shaded region and the atom sphere.

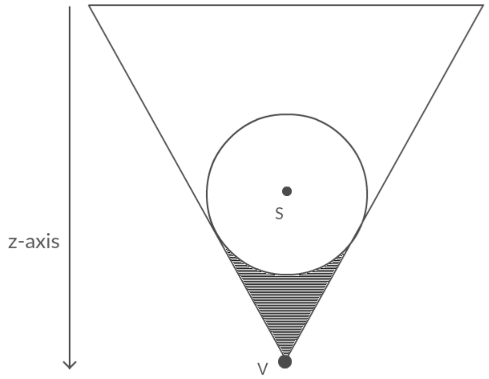

Figure 1: Schematics of the probe shape.

To determine the height of the atomic structure needed to generate simulated AFM images, we have to compute the hard collision of the tip shape with the atom spheres.

2 Collision between a cone and a sphere

We consider a cone with a vertex $V = (x_V, y_V, z_V)$ and a sphere of centre $S = (x_S, y_S, z_S)$ and radius r (see Figure 2). A point (x, y, z) on the surface of the cone is separated by a distance a from the central axis. The projection of this point to this axis is separated by a distance c from the vertex. We have $c = z_V - z$ and $a = (z_V - z)\tan(\alpha)$.
A point (x, y, z) on the surface of the sphere is separated by a distance b from the central axis and the projection of this point to this axis is separated by a distance d from the centre. We have $d = (z_S - z)$ and $b = \sqrt{r^2 - (z_S - z)^2}$.
We also consider the two dimensional distance f between the vertex V and the centre S, $f = \sqrt{(x_V - x_S)^2 + (y_V - y_S)^2}$.
For the collision point $I = (x, y, z)$, the collision condition is fulfilled (see Figure

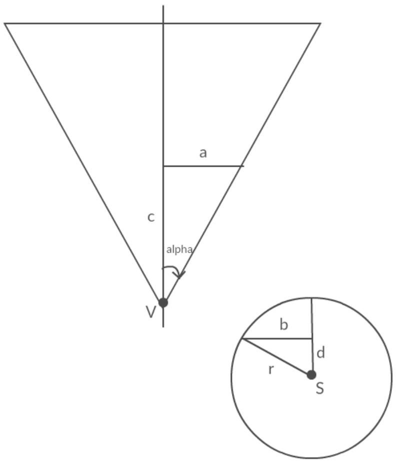

Figure 2: Schematics of cone and sphere with the used distances indicated.

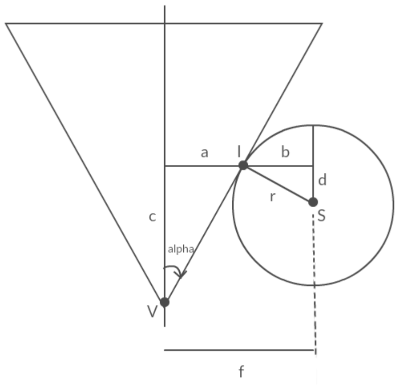

Figure 3: Schematics of a collision between the cone and the sphere.

3). It reads

$$a + b = f. \tag{1}$$

We have $(z_V - z)\tan(\alpha) + \sqrt{r^2 - (z_S - z)^2} = f$,

which leads to a second-order polynomial equation in z:

$$(1 + \tan^2(\alpha))z^2 + (-2z_V \tan^2(\alpha) + 2f\tan(\alpha) - 2z_S)z \\ + (f^2 - 2fz_V \tan(\alpha) + z_V^2 \tan^2(\alpha) - r^2 + z_S^2) = 0 \tag{2}$$

Depending on z_V this equation can have either no solution, i.e. no intersection (Figure 2), one unique solution(Figure 3), or 2 solutions (Figure 4).
The unique solution case corresponds to the collision where the cone is tangent to the sphere.

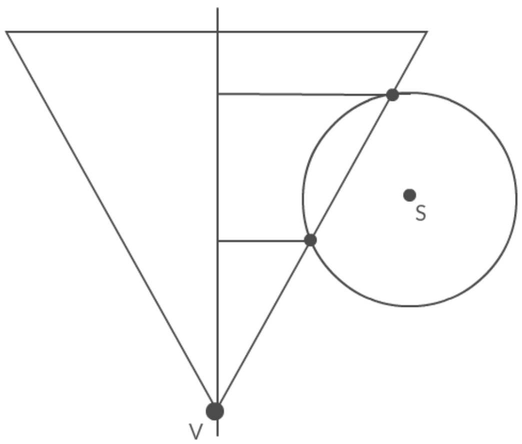

Figure 4: Schematic representation of the sphere penetrating the cone, see text for explanation.

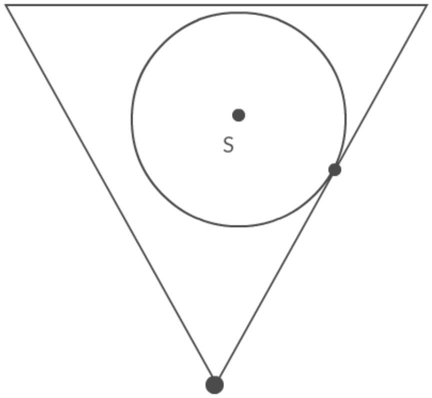

Figure 5: Schematics of cone and sphere collision, which is discarded. See text for explanation.

This corresponds to the vanishing discriminant Δ of the polynomial in equation (2), which will determine z_V. This gives a second order polynomial equation in z_V

$$(-\tan^2(\alpha))z_V^2 + (2z_S\tan^2(\alpha) + 2f\tan(\alpha))z_V \\ + (-2fz_S\tan(\alpha) - f^2 + r^2 + r^2\tan^2(\alpha) - z_S^2\tan^2(\alpha)) = 0. \quad (3)$$

The discriminant of the corresponding polynomial is $\Delta_2 = 4r^2\tan^2(\alpha)(\tan^2(\alpha)+1) > 0$.

Then, equation (3) has always two solutions. It means there are two positions of the vertex for which the equation (2) has a unique solution. They are shown in Figure 3 and Figure 5. The smaller solution z_V is taken.

The solutions are $z_V = \dfrac{z_S \tan(\alpha) + f \pm r\sqrt{\tan^2(\alpha)+1}}{\tan(\alpha)}$

and $z = \dfrac{z_V \tan^2(\alpha) - f\tan(\alpha) + z_S}{1+\tan^2(\alpha)}$.

3 Collision between two spheres

A sphere $S = (x_S, y_S, z_S)$ of radius r collides with a sphere $P = (x_P, y_P, z)$ of radius r_P (the probe sphere) if the sum of their radii is equal to the distance between their centres.
The collision condition is

$$f^2 + (z_S - z)^2 = (r + r_P)^2,$$

where $f = \sqrt{(x_P - x_S)^2 + (y_P - y_S)^2}$.

This given a second-order polynomial equation in z,

$$z^2 - 2z_s z + (f^2 - (r + r_P)^2 + z_S^2) = 0.$$

The discriminant is $\Delta = -4(f^2 - (r + r_P)^2)$.

The solutions are $z = z_S \pm \sqrt{(r + r_P)^2 - f^2}$. The smaller solution is taken.
