# Supplementary figures and images for: BioAFMviewer: An interactive interface for simulated AFM scanning of biomolecular structures and dynamics
Source: PLoS Comput Biol. 2020 Nov 18;16(11):e1008444. doi: 10.1371/journal.pcbi.1008444 (PMC7710046; doi:10.1371/journal.pcbi.1008444)

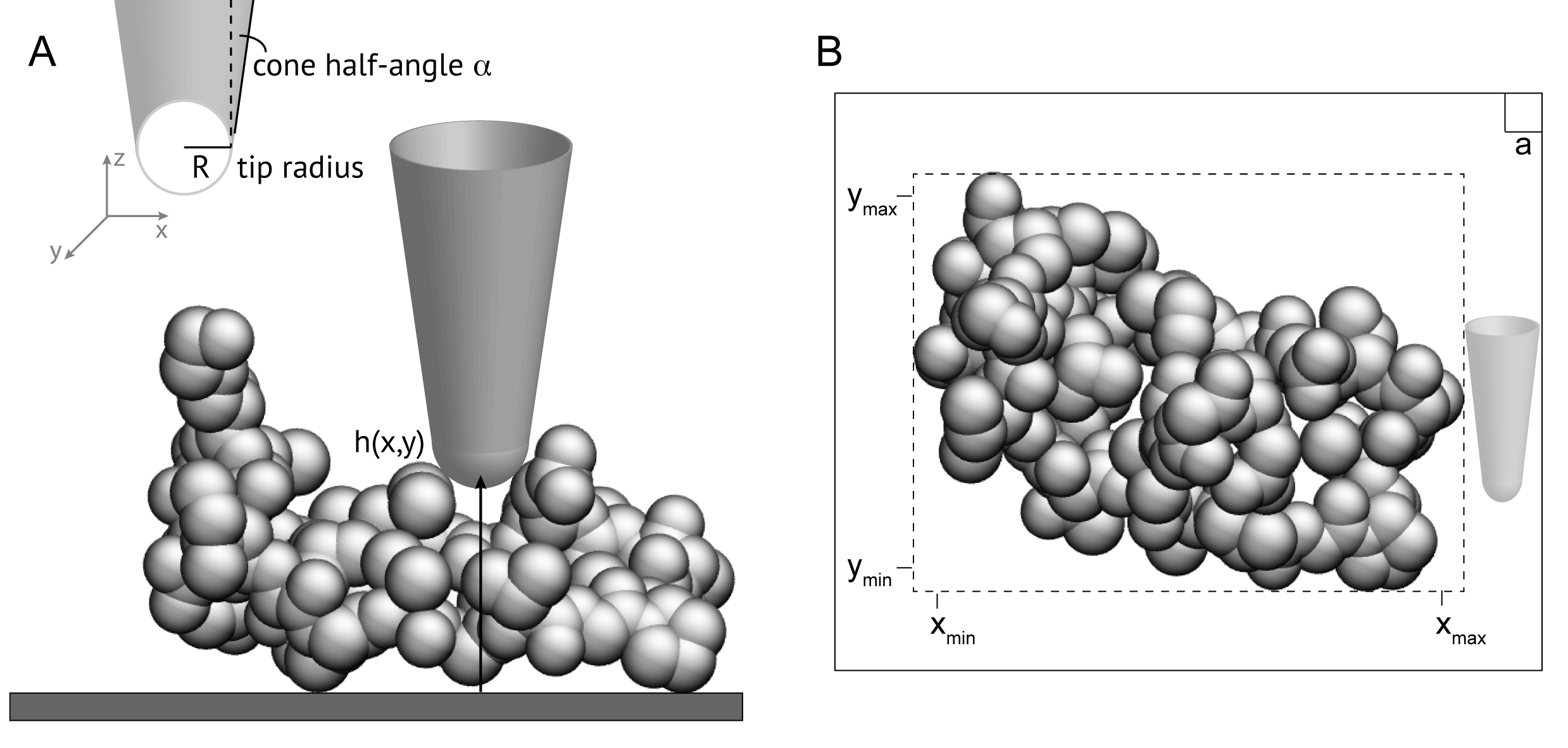

Supplement: S1 Fig — A) The cone-shaped tip with its geometric parameters and the tip-structure hard collision method to generate simulated AFM images are illustrated. The molecular structure is shown in the VdW representation and the virtual sample surface determined for the given scanning orientation is indicated by the dark gray bar. B) The size of the scanning area for a given scanning orientation is shown. The step size along the scanning grid is denoted by a. (TIF) [file pcbi.1008444.s002.tif]

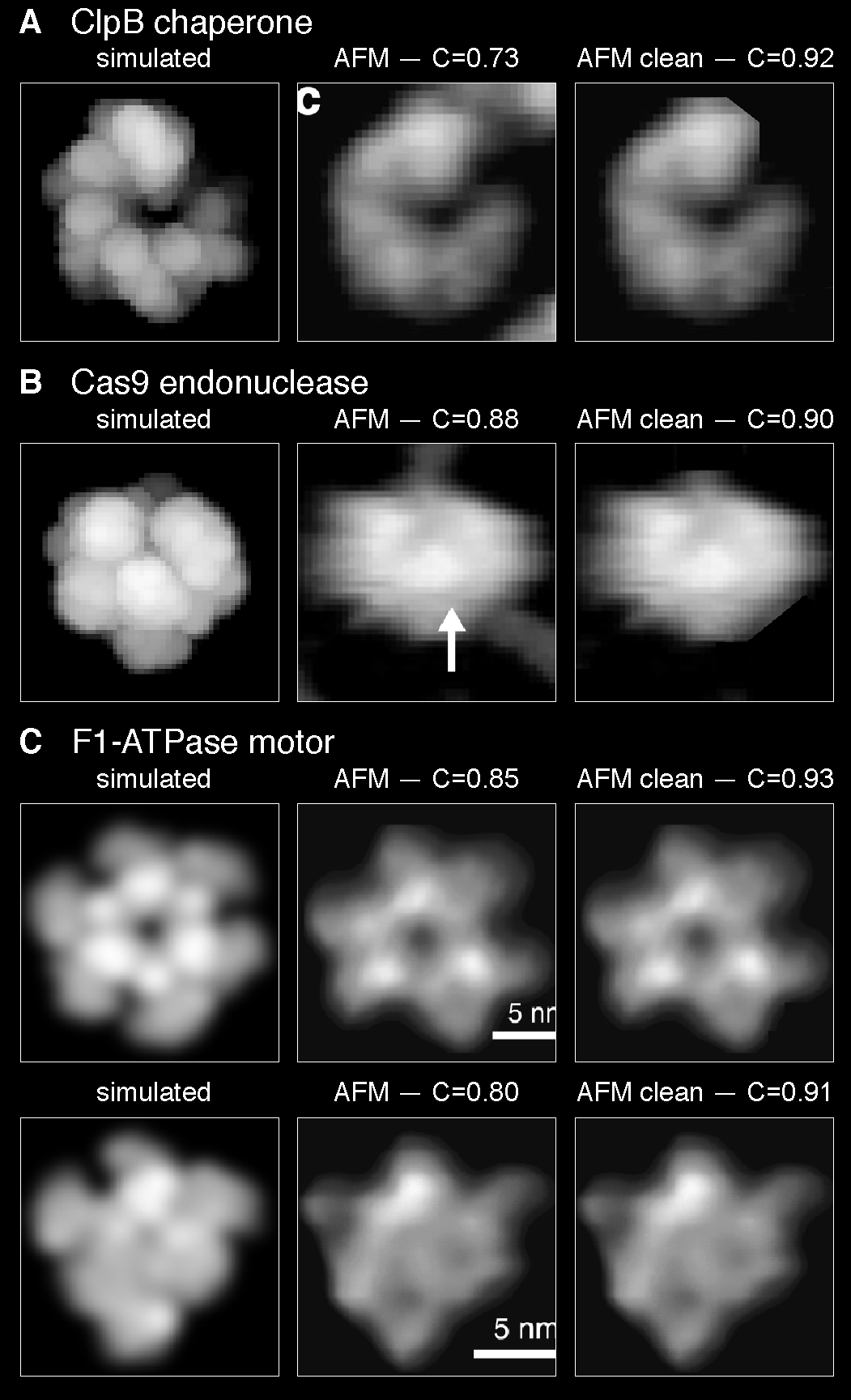

Supplement: S2 Fig — For the examples shown in main text Fig 3, the similarity between simulated and hs-AFM images is quantified by means of their pixel intensities. Only defined regions of interest are displayed in grayscale. Left images show the simulated AFM graphics, middle images the hs-AFM graphics, and right images provide corresponding cleaned versions for more realistic comparison. In all cases, the correlation value C of simulated and experimental images is given (see S1 Text). (TIF) [file pcbi.1008444.s003.tif]

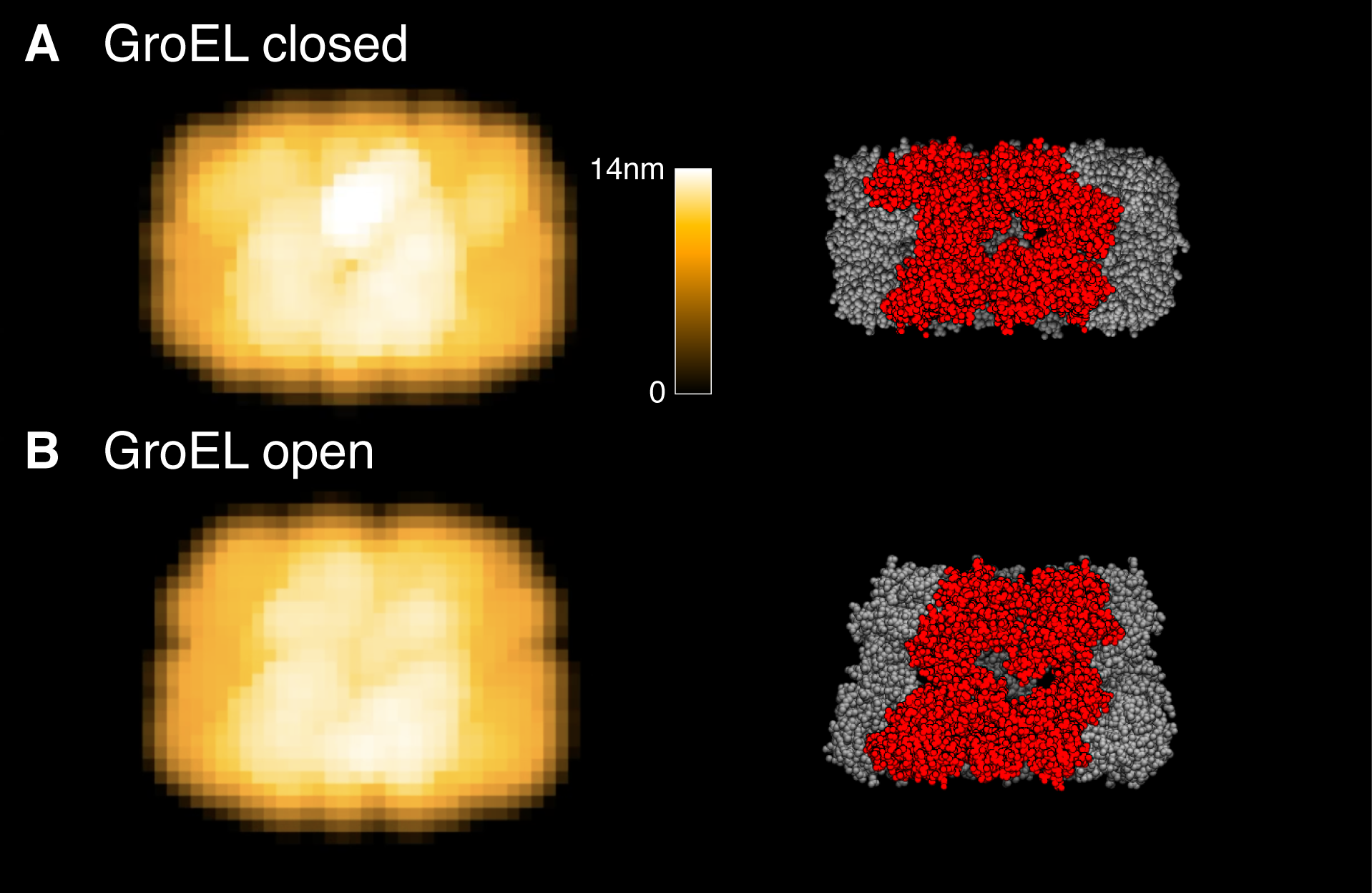

Supplement: S3 Fig — Snapshots from the molecular movie (S3 Video) of the GroEL complex in the closed and open state (A,B). Simulated AFM images and the corresponding molecular representation are shown side by side. In the VdW representation two adjacent domains in the 7-mer ring are highlighted by red color. The functional change in their shape is nicely resolved in the height protrusions of the simulated images. (TIF) [file pcbi.1008444.s004.tif]
